# Exploring the Eco-Evolutionary Dynamics of Tumor Subclones

**DOI:** 10.3390/cancers12113436

**Published:** 2020-11-19

**Authors:** Theodoros Rampias

**Affiliations:** Biomedical Research Foundation of the Academy of Athens, Basic Research Center, 11527 Athens, Greece; trampias@bioacademy.gr; Tel.: +30-210-659-7469

Mutational processes constantly shape the cancer genome and defects in DNA repair pathways of tumor cells facilitate the accumulation of genomic alterations. The advent of next-generation sequencing technology has contributed to a better understanding of the link between mutational processes and the generation of specific mutational signatures. Bioinformatic analysis of the mutational profile of 7000 tumors from over 30 cancer types has identified at least 20 distinct mutational signatures, which reflects the diverse nature of the endogenous and exogenous genome instability processes that are acting in cancers during evolution. For example, G∙C→T∙A transversions are characteristic of mutations associated with exposure to smoking-related DNA damage agents in lung cancers, whereas skin cancer mutations associated with ultraviolet (UV) radiation exposure are represented predominantly by C∙G→T∙A transitions. Similarly, post-replicative mismatch repair deficiency (MMRd) is represented by C∙G→T∙A transitions at an NpCpG sequence context and C∙G→A∙T transversions at CpCpC sequence context [1].

In precancerous lesions, oncogene activation by acquired mutations, increases the proliferation potential of precancerous cells and promotes replication stress. Collapse of stalled DNA replication forks can form microdeletions and gross chromosomal rearrangements that affect the genome integrity genes involved in DNA repair and cell cycle checkpoint regulation, leading to genomic instability. Increasing instability, in turn, contributes to accumulation of additional mutations that favor cancer development [2]. The acquired mutations can be categorized into those that do not provide any benefit to cancer progression and therefore are selectively neutral (passenger mutations), those that are disadvantageous for the cancer cell and therefore are subjected to negative selection (deleterious mutations), and those that increase survival or proliferation, conferring a selective advantage during tumor evolution (driver mutations). In addition to oncogene activation, other hallmarks linked to driver mutations are those of escape from apoptosis and senescence (e.g., TP53 mutations), the replication immortality (e.g., TERC mutations) and the induction of angiogenesis (VHL mutations) [3]. A tumor’s mutational profile represents a catalogue of alterations that have accumulated during its history. In clonal evolution models, genomic instability results in a stochastic accumulation of mutations that increase genetic diversity within the tumor and generate subclones with distinct genotypes over time. In this context, the intra tumoral heterogeneity can be used to identify the temporal order of acquired mutation acquisition [4]. Common alterations in analyzed subclones form the base of a cancer’s somatic evolutionary tree, while mutations that have subclonal origin, are used to form the branches. Further, the prevalence of subclonal mutations can be used to decode the subclonal hierarchy of the tumor’s phylogeny [5]. 

Genome instability processes can also cause chromosomal instability (CIN) which is associated with altered chromosome number (gain or loss of chromosomes) or structure (gain or deletion of chromosome fragments, translocations, inversions). Both numerical (aneuploidy) and structural CIN promote gene copy number alterations (CNAs) that affect transcriptome. Somatic CNAs in cancer include chromosomal regions that affect known oncogene or tumor suppressor genes. It is well established that specific mutations in DNA damage response/repair genes are associated with high levels of CNAs. For example, loss of function mutations of TP53 or mutations leading to homologous recombination (HR) deficiency are associated with allelic imbalance [6,7]. Genomic copy number heterogeneity can also be extensive within tumors while unstable aneuploidy has been shown to favor the generation of various tumor subpopulations leading to inter and intratumoral genomic heterogeneity [8]. It becomes clear that copy number variations should be taken under consideration together with mutation data when inferring the evolutionary history of tumors.

Tumors are also characterized by transcriptomic and immunogenic heterogeneity. Besides the effect of mutations and gene copy number alterations on transcriptome profile, a wide variation of epigenetic mechanisms including DNA methylation, chromatin remodeling and histone modifications, contribute to gene expression diversity within tumors [9]. Transcriptomic heterogeneity is also generated through cellular differentiation of cancer stem cell populations or through the selective pressure on distinct cancer stem cell subgroups [10]. Single cell RNA-seq (scRNA-seq) technology is a useful tool for the unbiased identification of different transcriptional programs within the tumor and therefore for the molecular subtyping of neoplastic clones with different expression profiles. In this direction, the expression of cancer stem cell or differentiation markers in scRNA-seq analysis can help us identify cancer stem cell populations and early differentiated subpopulations within the tumor [11].

Growing tumors are initially subjected to a strong immunoediting process, as the immune system eliminates cancer cells recognizing neoantigens on their surface. During tumor evolution, immune escape mechanisms are established favoring the emergence of new subclones that are no longer recognized by adaptive immunity or promote immunosuppression within the tumor microenvironment. By this way, cancer immunoediting increases tumor heterogeneity [12]. As immunoediting functions based on intercellular contacts and secretion of cytokines and chemokines, the spatial profile of interactions between the tumor immune microenvironment and the neoplastic clones determines the intensity of pressure in individual clones. Tumor infiltration and spatial distribution of CD8+ T cells in tumor invasive front and stroma have been recognized as prognostic factors in different cancers. For instance, high levels of T-cell infiltration in intratumor regions but not in the peritumoral stroma are associated with increased survival in hormone receptor negative breast cancer [13], ovarian cancer [14], colorectal cancer [15] and urothelial carcinoma [16]. Spatial distribution of other immune components, such as cancer-associated fibroblasts and tertiary lymphoid structures in tumor architecture, has also emerged as an important prognostic factor, highlighting the need for mapping the spatial context and subtyping the molecular profile of immune microenvironment. In the near future, spatial analysis enforced by advanced imaging analysis and the use of learning machine could facilitate studies of ecological interactions between tumor and immune subpopulations, helping with the identification of patients at higher risk of progression or treatment resistance.

The need of tumor cells for sufficient supply of nutrients and oxygen represents another major microenvironmental selection pressure to cancer cells [17]. Thus, tumor subclones that are metabolically adapted to survive in hypoxic conditions or stimulate angiogenesis are able to survive this pressure, and become selected. Most solid tumors exhibit spatial heterogeneity (tumor center vs. tumor periphery) regarding the tumor oxygenation while temporal waves of hypoxia are also common. For instance, acute hypoxia can result from increased interstitial fluid pressure [18], while the upregulation of vascular endothelial growth factor (VEGF) can lead to increased angiogenesis and reoxygenation of hypoxic regions [19]. An adaptive response of cancer cells to hypoxia is mediated through the stabilization of hypoxia inducible factor (HIF), which increases the transcription of several genes mediating the response to hypoxia [20]. A synopsis of selective pressures by tumor microenvironment is presented in Figure 1.

Since, the interaction between tumor subclones and the surrounding normal tissue and immune microenvironment plays a vital role in clonal selection, the spatial distribution of cancer subclones in their microenvironment is an important parameter for the pressure intensity and the clone selection dynamics. For instance, the tumor invasive front is considered to be enriched in cancer stem cell populations in different tumor types [21,22,23]. Moreover, many invasive signaling pathways overlap with known cancer stem cell expression signatures. For example, expression of cancer stem cell markers CD44, CXCR4, ALDH1A3 and epithelial–mesenchymal transition (EMT) signatures are prevalent at the invasive front in a wide variety of cancers [24,25,26].

Regarding the population dynamics that operate within heterogeneous tumors, the vast majority of subclonal interactions are neutral. However, clonal competition for space and nutrients can eventually result in clonal selection andsurvival of the fittest, according to Darwinian model of evolution. On the other hand, collaborative interactions among clones have also been described and are considered to contribute on tumor heterogeneity and cancer progression. For example, paracrine secretion of growth factors from one clone can sustain the survival and proliferation of other clones (Figure 2).

Importantly, the interaction between the multiple layers of mutational, transcriptomic and immunogenic heterogeneity creates a complex ecosystem that drives multistep processes in tumor evolution such as metastasis and drug resistance. Metastasis requires local invasion, intravasation, survival in the circulation, extravasation and distant colonization. Each of these steps relies on specific oncogenic alterations and interactions with the host microenvironment and the immune system. Recent sequencing studies in cohorts of paired primary and metastatic tumors have provided unprecedented biological insights in metastatic process. Metastases are generally characterized by a reduction in the number of mutations compared to primary tumor, indicating a monoclonal pattern of seeding in the vast majority of metastases. However, polyclonal seeding has also been observed, providing evidence that the initial clone can remodel the metastatic site, making it attractive for additional clones to colonize [27]. Two progression models for metastasis have been proposed from comparative analysis of primary tumor subclonal complexity and the mutation profile of paired metastatic tumor. According to the linear progression model, metastasis is seeded at a late stage of tumor progression, resulting in minimal genetic divergence between the primary and metastatic tumor. Conversely, in the parallel progression model, metastatic dissemination emerges early in tumor progression, and both the primary and metastatic tumor evolve in parallel, resulting in substantial genetic divergence [28]. In recent years, there has been an increasing development of liquid biopsy as a non-invasive and rapid approach for monitoring of the clonal dynamics of primary and metastatic tumor [29]. However, since metastasis is the primary cause of death for many cancer patients, it has become clear that for a better understanding of clonal dynamics on metastasis, further and larger-scaled prospective studies on cohorts with paired primary and metastatic tumors are required. While many cancer-driver genes have been identified through large-scale sequencing efforts, to date, the catalogue of driver mutations that are strongly associated with metastasis is limited. There is compelling evidence for the association of TP53 mutations with metastasis in specific types solid tumors. In metastatic breast cancer, TP53 mutations, as well as mutations in ESR1, NF1, AKT1, KMT2C and PTEN genes, were found enriched in ER+/HER2− metastatic lesions [30]. Similarly, in head and neck cancer, specific TP53 mutations in its DNA binding interface are enriched in metastatic tumors compared to primary ones [31]. Finally, there is increasing evidence that specific TP53 missense mutations with dominant-negative or gain-of-function (GOF) activity are associated with metastasis in breast, lung and prostate cancer [32,33].

Traditional treatments such as chemotherapy and radiation rely on the induction of DNA damage, which is more cytotoxic for proliferating cells, exploiting the vulnerability of cancer cells to DNA damage repair. Despite the fact that chemotherapy and radiotherapy have been used in clinics for many decades, intrinsic (present at baseline) or acquired (developed after initial treatment) resistance during treatment cycles and high toxicity in patients severely limit the clinical benefit. Numerous studies have shown that intratumor heterogeneity drives drug resistance and therefore poses a major challenge in the efficient curing of cancer patients. Any given treatment applied to a heterogeneous tumor will yield distinct responses in different clones and may be ineffective in eliminating the resistant ones. Moreover, upon treatment, in highly heterogeneous tumors, stochastically, there is a higher chance of acquisition and selection of mutations that drive drug resistance. Therefore, outgrowth of resistant clones (whether pre-existing or acquired de novo) under the selective pressure, leads to therapeutic failure [34]. For example, intrinsic or acquired KRAS or HRAS mutations confer resistance to EGFR targeting therapy in colorectal and head and neck cancer [35]. Similarly, RAF amplification or MEK1 mutation has been shown to drive resistance to BRAF inhibitors in V600E mutant melanoma [36] and in BRCA-mutant tumors, specific intragenic deletions or revertant mutations in BRCA1/2 genes can restore their activity on DNA repair pathways promoting resistance to PARP inhibitors [37,38].

It has been increasingly recognized over the past decade that intra-tumor heterogeneity is associated with the process of clonal evolution and that cancer progression can be interpreted within an ecological and evolutionary framework. In the era of personalized medicine, there is an urgent need for this concept to be transformed into clinical practice, and define the impact of intra-tumor heterogeneity on drug response and metastasis.

Since intrinsic and acquired resistance to treatment is frequently related to specific genetic alterations, sequencing studies that include sequential time-ordered sampling of cancers at baseline, during and after treatment, are needed in order to catalogue mutations on tumor subclones that trigger intrinsic or acquired drug resistance.

The emergence of immunotherapy has transformed the clinical landscape for the treatment of patients with advanced or metastatic cancer, and tumor mutation burden (TMB) has been recognized as an emerging biomarker for response to immune checkpoint inhibitors [39]. Interestingly, recent studies have shown that both the number of subclones within the tumor and the degree of their genetic diversity can also determine the efficiency of immunotherapy and that patients with tumors with high levels of heterogeneity show poorer survival. As a result, intratumoral heterogeneity (ITH) has been proposed as a better predictive biomarker for immunotherapy compared to tumor mutation burden (TMB) [40]. High levels of ITH are known to promote the outgrowth of immune escape clones. Moreover, it has been also proposed that high ITH is associated with a weaker representation of clone-specific neoantigens that drive immune infiltration, within the tumor surface [40].

Synthetic lethality provides a new approach for the treatment of tumors with mutated DNA damage response genes that were previously considered unable to be targeted in traditional targeted treatments. DNA damage deficiency in these tumors is strongly associated with genomic instability and high levels of replication stress and heterogeneity. In this context, PARP and PARG inhibitors exploit these tumor vulnerabilities by inducing further DNA damage, preventing DNA repair and promoting mitotic catastrophe [41]. The increasing applications of PARP inhibitors in the clinical setting for the treatment of BRCA1/BRCA2-deficient breast and ovarian cancers, made synthetic lethality a promising anticancer treatment option [42].

Compared with traditional sequencing technology, single-cell sequencing (SC-sec) has the advantage of detecting transcriptomic (scRNA-sec) or genomic (scDNA-seq) heterogeneity among individual cells. However, the current use of SC-seq in clinical practice is limited due to its high cost.

In the near future, we expect that progress in scDNA-seq technology will help us to measure ITH and DDR mutations in clonal populations of tumor samples with low cost, in order to predict the clinical response to immunotherapy and DNA repair inhibitors [43].

Metastatic disease remains largely incurable despite the progress in drug development. Despite the limitations on biopsying the metastatic site, it is apparent that over the next few years, new sequencing studies will provide deeper insight into the phylogenetic relationships between primary and metastatic tumors. A better understanding of the evolutionary processes that contribute to the emergence of metastatic subclones will reveal the therapeutic vulnerabilities in metastatic tumors.

Recent technological advances have resulted in greatly improved detection and culture of circulating tumor cells. Nowadays, liquid biopsy has been approved for ctDNA analysis in Europe for patients with non-small-cell lung carcinoma for EGFR mutation testing [44]. However, the application of liquid biopsy to clinical practice is still limited. In the near future, we can expect that progress in technologies related to isolation of circulating cancer cells and single cell mutational profiling will help us to monitor treatment efficacy and clonal evolution in relation to treatment resistance and cancer metastasis, with minimally invasive methods.

## Figures and Tables

**Figure 1:**
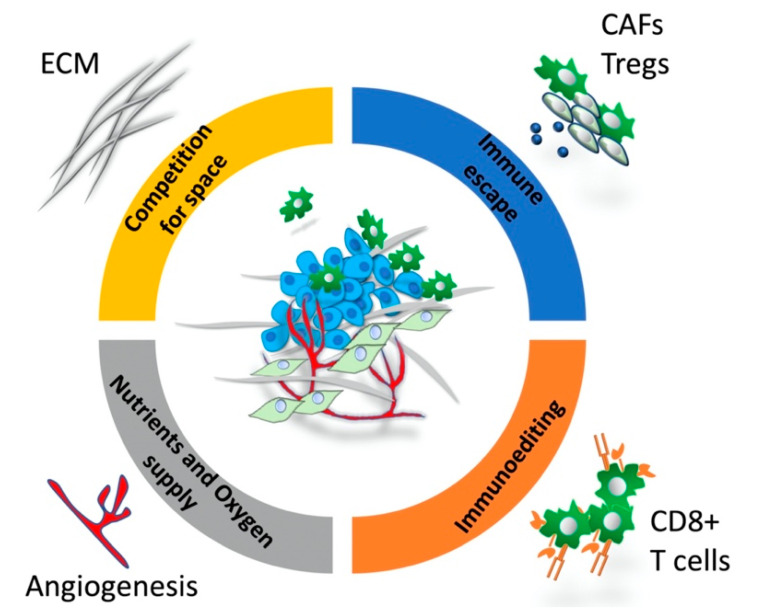
Tumor microenvironment and evolutionary pressures on cancer populations. ECM: extracellular matrix, CAFs: cancer-associated fibroblasts, Tregs: regulatory T cells, CD8+ T cells: cytotoxic T cells.

**Figure 2:**
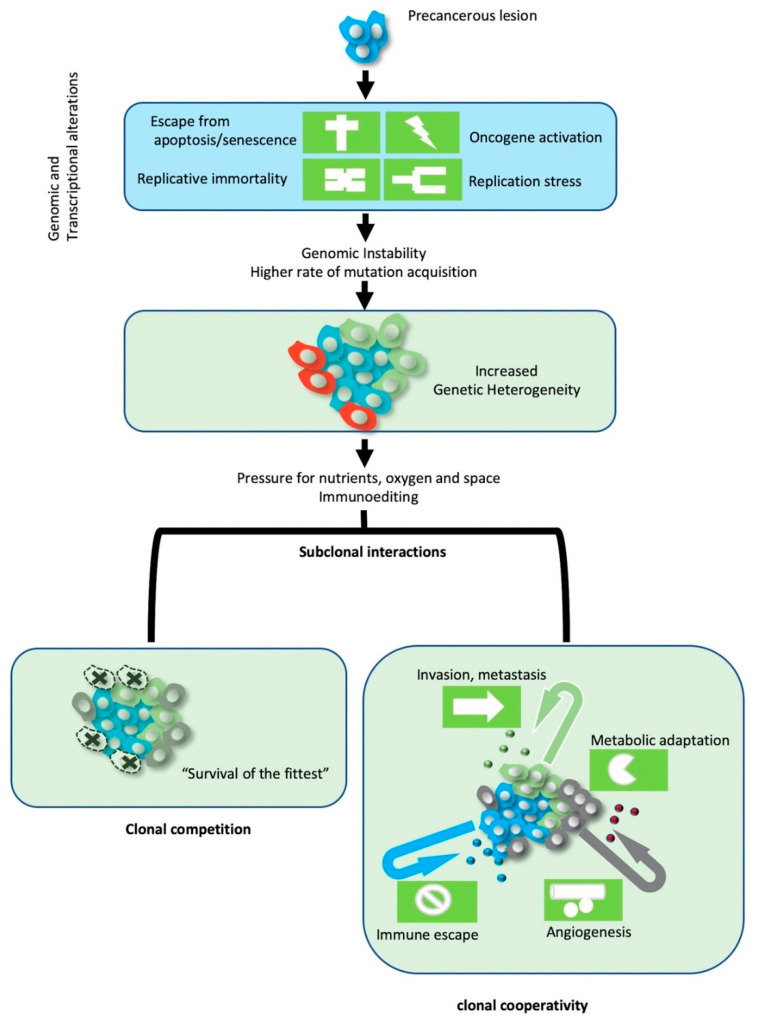
Tumor heterogeneity and clonal interactions. Oncogene-induced replication stress drives genomic instability and clonal heterogeneity within tumors. Selective pressures and complex subclonal interactions within the tumor ecosystem drive the evolutionary and ecological features of growing tumor.

## References

[1] Alexandrov L.B., Nik-Zainal S., Wedge D.C., Aparicio S.A., Behjati S., Biankin A.V., Bignell G.R., Bolli N., Borg A., Borresen-Dale A.L. (2013). Signatures of mutational processes in human cancer. Nature.

[2] Halazonetis T.D., Gorgoulis V.G., Bartek J. (2008). An oncogene-induced DNA damage model for cancer development. Science.

[3] Macheret M., Halazonetis T.D. (2015). DNA replication stress as a hallmark of cancer. Annu. Rev. Pathol..

[4] McGranahan N., Swanton C. (2017). Clonal Heterogeneity and Tumor Evolution: Past, Present, and the Future. Cell.

[5] Schwartz R., Schaffer A.A. (2017). The evolution of tumour phylogenetics: Principles and practice. Nat. Rev. Genet..

[6] Donehower L.A., Soussi T., Korkut A., Liu Y., Schultz A., Cardenas M., Li X., Babur O., Hsu T.K., Lichtarge O. (2019). Integrated Analysis of TP53 Gene and Pathway Alterations in The Cancer Genome Atlas. Cell Rep..

[7] Macintyre G., Goranova T.E., De Silva D., Ennis D., Piskorz A.M., Eldridge M., Sie D., Lewsley L.A., Hanif A., Wilson C. (2018). Copy number signatures and mutational processes in ovarian carcinoma. Nat. Genet..

[8] Gagos S., Irminger-Finger I. (2005). Chromosome instability in neoplasia: Chaotic roots to continuous growth. Int. J. Biochem. Cell Biol..

[9] Flavahan W.A., Gaskell E., Bernstein B.E. (2017). Epigenetic plasticity and the hallmarks of cancer. Science.

[10] La Porta C.A.M., Zapperi S. (2017). Complexity in cancer stem cells and tumor evolution: Toward precision medicine. Semin. Cancer Biol..

[11] Gonzalez-Silva L., Quevedo L., Varela I. (2020). Tumor Functional Heterogeneity Unraveled by scRNA-seq Technologies. Trends Cancer.

[12] Schreiber R.D., Old L.J., Smyth M.J. (2011). Cancer immunoediting: Integrating immunity’s roles in cancer suppression and promotion. Science.

[13] Li X., Gruosso T., Zuo D., Omeroglu A., Meterissian S., Guiot M.C., Salazar A., Park M., Levine H. (2019). Infiltration of CD8(+) T cells into tumor cell clusters in triple-negative breast cancer. Proc. Natl. Acad. Sci. USA.

[14] Zhang L., Conejo-Garcia J.R., Katsaros D., Gimotty P.A., Massobrio M., Regnani G., Makrigiannakis A., Gray H., Schlienger K., Liebman M.N. (2003). Intratumoral T cells, recurrence, and survival in epithelial ovarian cancer. N. Engl. J. Med..

[15] Galon J., Costes A., Sanchez-Cabo F., Kirilovsky A., Mlecnik B., Lagorce-Pages C., Tosolini M., Camus M., Berger A., Wind P. (2006). Type, density, and location of immune cells within human colorectal tumors predict clinical outcome. Science.

[16] Sharma P., Shen Y., Wen S., Yamada S., Jungbluth A.A., Gnjatic S., Bajorin D.F., Reuter V.E., Herr H., Old L.J. (2007). CD8 tumor-infiltrating lymphocytes are predictive of survival in muscle-invasive urothelial carcinoma. Proc. Natl. Acad. Sci. USA.

[17] Graeber T.G., Osmanian C., Jacks T., Housman D.E., Koch C.J., Lowe S.W., Giaccia A.J. (1996). Hypoxia-mediated selection of cells with diminished apoptotic potential in solid tumours. Nature.

[18] Heldin C.H., Rubin K., Pietras K., Ostman A. (2004). High interstitial fluid pressure—An obstacle in cancer therapy. Nat. Rev. Cancer.

[19] Jain R.K. (2005). Normalization of tumor vasculature: An emerging concept in antiangiogenic therapy. Science.

[20] Kim J.W., Tchernyshyov I., Semenza G.L., Dang C.V. (2006). HIF-1-mediated expression of pyruvate dehydrogenase kinase: A metabolic switch required for cellular adaptation to hypoxia. Cell Metab..

[21] Kodama H., Murata S., Ishida M., Yamamoto H., Yamaguchi T., Kaida S., Miyake T., Takebayashi K., Kushima R., Tani M. (2017). Prognostic impact of CD44-positive cancer stem-like cells at the invasive front of gastric cancer. Br. J. Cancer.

[22] Yoshida G.J. (2017). The heterogeneity of cancer stem-like cells at the invasive front. Cancer Cell Int..

[23] Luo W.R., Yao K.T. (2014). Cancer stem cell characteristics, ALDH1 expression in the invasive front of nasopharyngeal carcinoma. Virchows Arch..

[24] Marcato P., Dean C.A., Pan D., Araslanova R., Gillis M., Joshi M., Helyer L., Pan L., Leidal A., Gujar S. (2011). Aldehyde dehydrogenase activity of breast cancer stem cells is primarily due to isoform ALDH1A3 and its expression is predictive of metastasis. Stem Cells.

[25] Reuben J.M., Lee B.N., Gao H., Cohen E.N., Mego M., Giordano A., Wang X., Lodhi A., Krishnamurthy S., Hortobagyi G.N. (2011). Primary breast cancer patients with high risk clinicopathologic features have high percentages of bone marrow epithelial cells with ALDH activity and CD44(+)CD24lo cancer stem cell phenotype. Eur. J. Cancer.

[26] Costa L.C., Leite C.F., Cardoso S.V., Loyola A.M., Faria P.R., Souza P.E., Horta M.C. (2015). Expression of epithelial-mesenchymal transition markers at the invasive front of oral squamous cell carcinoma. J. Appl. Oral Sci..

[27] Turajlic S., Swanton C. (2016). Metastasis as an evolutionary process. Science.

[28] Zhao Z.M., Zhao B., Bai Y., Iamarino A., Gaffney S.G., Schlessinger J., Lifton R.P., Rimm D.L., Townsend J.P. (2016). Early and multiple origins of metastatic lineages within primary tumors. Proc. Natl. Acad. Sci. USA.

[29] Russano M., Napolitano A., Ribelli G., Iuliani M., Simonetti S., Citarella F., Pantano F., Dell’Aquila E., Anesi C., Silvestris N. (2020). Liquid biopsy and tumor heterogeneity in metastatic solid tumors: The potentiality of blood samples. J. Exp. Clin. Cancer Res..

[30] Angus L., Smid M., Wilting S.M., van Riet J., Van Hoeck A., Nguyen L., Nik-Zainal S., Steenbruggen T.G., Tjan-Heijnen V.C.G., Labots M. (2019). The genomic landscape of metastatic breast cancer highlights changes in mutation and signature frequencies. Nat. Genet..

[31] Klinakis A., Rampias T. (2020). TP53 mutational landscape of metastatic head and neck cancer reveals patterns of mutation selection. EBioMedicine.

[32] Milner J., Medcalf E.A. (1991). Cotranslation of activated mutant p53 with wild type drives the wild-type p53 protein into the mutant conformation. Cell.

[33] Roszkowska K.A., Gizinski S., Sady M., Gajewski Z., Olszewski M.B. (2020). Gain-of-Function Mutations in p53 in Cancer Invasiveness and Metastasis. Int. J. Mol. Sci..

[34] Burrell R.A., Swanton C. (2014). Tumour heterogeneity and the evolution of polyclonal drug resistance. Mol. Oncol..

[35] Misale S., Di Nicolantonio F., Sartore-Bianchi A., Siena S., Bardelli A. (2014). Resistance to anti-EGFR therapy in colorectal cancer: From heterogeneity to convergent evolution. Cancer Discov..

[36] Lito P., Rosen N., Solit D.B. (2013). Tumor adaptation and resistance to RAF inhibitors. Nat. Med..

[37] Johnson N., Johnson S.F., Yao W., Li Y.C., Choi Y.E., Bernhardy A.J., Wang Y., Capelletti M., Sarosiek K.A., Moreau L.A. (2013). Stabilization of mutant BRCA1 protein confers PARP inhibitor and platinum resistance. Proc. Natl. Acad. Sci. USA.

[38] Bouwman P., Jonkers J. (2014). Molecular pathways: How can BRCA-mutated tumors become resistant to PARP inhibitors?. Clin. Cancer Res..

[39] Chan T.A., Yarchoan M., Jaffee E., Swanton C., Quezada S.A., Stenzinger A., Peters S. (2019). Development of tumor mutation burden as an immunotherapy biomarker: Utility for the oncology clinic. Ann. Oncol..

[40] Wolf Y., Bartok O., Patkar S., Eli G.B., Cohen S., Litchfield K., Levy R., Jimenez-Sanchez A., Trabish S., Lee J.S. (2019). UVB-Induced Tumor Heterogeneity Diminishes Immune Response in Melanoma. Cell.

[41] Slade D. (2020). PARP and PARG inhibitors in cancer treatment. Genes Dev..

[42] Bryant H.E., Schultz N., Thomas H.D., Parker K.M., Flower D., Lopez E., Kyle S., Meuth M., Curtin N.J., Helleday T. (2005). Specific killing of BRCA2-deficient tumours with inhibitors of poly(ADP-ribose) polymerase. Nature.

[43] Zhang J., Wang W., Huang J., Wang X., Zeng Y. (2020). How far is single-cell sequencing from clinical application?. Clin. Transl. Med..

[44] Remon J., Lacroix L., Jovelet C., Caramella C., Howarth K., Plagnol V., Rosenfeld N., Morris C., Mezquita L., Pannet C. (2019). Real-World Utility of an Amplicon-Based Next-Generation Sequencing Liquid Biopsy for Broad Molecular Profiling in Patients With Advanced Non-Small-Cell Lung Cancer. JCO Precis. Oncol..

